# Neuromodulators as Interdomain Signaling Molecules Capable of Occupying Effector Binding Sites in Bacterial Transcription Factors

**DOI:** 10.3390/ijms242115863

**Published:** 2023-11-01

**Authors:** Yuri A. Purtov, Olga N. Ozoline

**Affiliations:** Department of Functional Genomics of Prokaryotes, Institute of Cell Biophysics of the Russian Academy of Sciences, Federal Research Center Pushchino Scientific Center for Biological Research of the Russian Academy of Sciences, Pushchino 142290, Russia

**Keywords:** neuromodulators, hormones, neurotransmitters, microbiomes, transcription factor–effector interaction, flexible molecular docking

## Abstract

Hormones and neurotransmitters are important components of inter-kingdom signaling systems that ensure the coexistence of eukaryotes with their microbial community. Their ability to affect bacterial physiology, metabolism, and gene expression was evidenced by various experimental approaches, but direct penetration into bacteria has only recently been reported. This opened the possibility of considering neuromodulators as potential effectors of bacterial ligand-dependent regulatory proteins. Here, we assessed the validity of this assumption for the neurotransmitters epinephrine, dopamine, and norepinephrine and two hormones (melatonin and serotonin). Using flexible molecular docking for transcription factors with ligand-dependent activity, we assessed the ability of neuromodulators to occupy their effector binding sites. For many transcription factors, including the global regulator of carbohydrate metabolism, CRP, and the key regulator of lactose assimilation, LacI, this ability was predicted based on the analysis of several 3D models. By occupying the ligand binding site, neuromodulators can sterically hinder the interaction of the target proteins with the natural effectors or even replace them. The data obtained suggest that the direct modulation of the activity of at least some bacterial transcriptional factors by neuromodulators is possible. Therefore, the natural hormonal background may be a factor that preadapts bacteria to the habitat through direct perception of host signaling molecules.

## 1. Introduction

The close coexistence of prokaryotes and eukaryotes over the long history of their co-evolution has developed a wide range of interdomain relationships, from symbiosis/mutualism to parasitism. At the molecular level, this has led to the emergence of multiple signaling systems perceived by both interacting parties. Microbial endocrinology, which comprehensively studies such inter-kingdom signaling systems, is based on the idea that symbiont interactions can be mediated by hormones and neurotransmitters, natural components of the humoral and neural regulatory systems of eukaryotes [1,2,3,4]. By recognizing signaling molecules in their environment, microbes can adapt their gene expression profile for successful colonization [5,6]. Both neurotransmitters [2,6,7,8] and hormones [9,10] were found in varying amounts in the intestinal lumen of vertebrates, the hormonal background of which is collectively created by the cells of the intestinal mucosa [11] and a microbial consortium that synthesizes and secretes a variety of bioactive molecules [12,13,14].

Evidence is gradually accumulating that suggests that most signaling molecules produced by mammalian hosts are capable of influencing the persistence of bacteria in the intestine [8,15,16,17]. Although most such data are obtained for the catecholamine neurotransmitters dopamine, epinephrine, and norepinephrine (derivatives of L-tyrosine), the hormones serotonin and melatonin (derivatives of tryptophan) are also considered important signaling molecules in host–microbiome interaction [18,19,20]. The concept of a bidirectional flow of signals between eukaryotes and microbes, mediated by neuroendocrine factors, was formulated in 1992, when M. Lyte and S. Ernst discovered the effects of norepinephrine on the growth of *Escherichia coli* (*E. coli*), *Yersinia enterocolitica*, and *Pseudomonas aeruginosa* [21]. Subsequently, the growth dependence on catheholamines was confirmed for other species [22,23,24,25], and it turned out that mammalian neuromodulators (NMs) can affect many physiological and metabolic properties of bacteria. The administration of norepinephrine to toxigenic and hemorrhagic strains of *E. coli*, for instance, stimulated the production of virulence factors [26], including Shiga toxins [27] and bacterial adherence to the colonic mucosa [28], while epinephrine and norepinephrine increased *E. coli* O157:H7 (EHEC) motility and biofilm formation [29]. Exogenous norepinephrine increased the cytotoxicity of *Vibrio parahaemolyticus* [30] and stimulated the ability of *Salmonella enterica* to colonize the host intestine [31], while melatonin improved the circadian rhythms of the intestinal microbiota of mice [16]. This diversity in the functional manifestations of NMs indicates that agents of the host hormonal systems can shape the microbiome by affecting several metabolic pathways in different bacteria. The gut microbiota, in turn, can also “shape gut physiology” [32], producing compounds that modulate host metabolism [18,20], including the biosynthesis of host hormones [18,20].

The identified changes in bacterial physiology are inevitably mediated by adaptive alterations in the expression of bacterial genes. However, currently, there are only a few data on NM-mediated changes in bacterial transcriptomes [29,33], and the available information mainly comes from studies on the effects caused by catecholamines on gene expression or the proteomes of pathogenic bacteria. Overall, it indicates that their administration causes alteration for a relatively large number of bacterial genes (0.6% in response to epinephrine in *Salmonella enterica* [34]), encoding pathogenicity factors, stress response proteins, and proteins involved in host colonization [35,36,37]. In the mutant EHEC strain with suppressed synthesis of the autoinducer AI-2, epinephrine activated the expression of the “enterocyte effacement” locus (LEE) in the pathogenicity island, playing a key role in the virulence of this strain [38,39]. In wild-type cells of EHEC, epinephrine and norepinephrine, along with virulence genes encoding proteins of chemotaxis, motility, biofilm formation, and colonization, enhanced the transcription of iron uptake genes and changed the expression of four transcription factors [29], of which LsrR pertains to the bacterial quorum sensing system. The pathogenic bacterium *Campylobacter jejuni*, exposed to the same NMs, altered several cellular functions, including virulence, motility, iron uptake, and the response to oxidative stress [37], while the norepinephrine-mediated cytotoxicity of *V. parahaemolyticus* appeared due to changes in the expression of the secretion system genes [31]. Finally, norepinephrine and dopamine stimulated the transcription of genes involved in the flagellar biogenesis and chemotaxis of *Vibrio harveyi* [40]. All this indicates a predictable “aggressive” reaction of pathogenic bacteria to the appearance of “stress hormones”. However, the decrease in the expression of *P. aeruginosa* motility genes in the presence of 50 μM norepinephrine was replaced by the stimulation of their transcription in response to a higher dose of the drug (500 μM) [41]. This dose dependence suggests a bimodal mechanism of regulatory signal transmission, which can be provided via the direct effect of norepinephrine on the expression of bacterial genes.

Toulouse et al. [42] and Scardaci et al. [43] studied the effect of catecholamines on the proteomes of pathogenic *Vibrio cholerae* and probiotic *Enterococcus faecium*, respectively. In *V. cholerae,* a set of 18 proteins with altered abundance included metabolic enzymes, iron transport, and homeostasis proteins, as well as the stress response protein UspA [42]. In *E. faecium*, along with transporters, stress-induced proteins, and transcription factor PhoU, the most reactive group, included several “host-interaction” proteins, such as bile salt hydrolase (BSH) and secreted antigen A (SagA) [43], which is consistent with the concept of an adaptive role of neuromodulators.

The modes of NMs’ action have so far been discussed only for catecholamines. It was discovered that in many Gram-negative bacteria, including EHEC, norepinephrine promotes the synthesis of a quorum-sensing inducer, AI-2 [44,45], which is unique in its ability to stimulate the growth of bacteria of its own and other species [45]. Synthesized by the highly conserved S-ribosylhomocysteine lyase LuxS, AI-2 has four types of membrane receptors in bacteria [46]. Consequently, the receptor-mediated NM signaling pathway has been addressed in several studies. Clark et al. [47] showed that norepinephrine and epinephrine interact with the QseC sensory kinase of the two-component QseBC quorum sensing system of EHEC. Therefore, the authors proposed that QseC is a bacterial receptor for catecholamines. Another two-component system potentially sensitive to the appearance of epinephrine and norepinephrine (QseEF) was also found in EHEC [48,49], while in *Salmonella,* this role may be played by the BasSR [34] and CpxAR [50] signal transduction systems. However, there are data that cast doubt on the function of QseC as a catecholamine receptor. The mutant strains of EHEC and *S. enterica* Typhimurium without genes encoding QseC and QseE retained the ability to respond to stress hormones [51,52], as well as some species, such as *Yersinia enterocolitica* lacking *qseC* [53]. Luqman et al. reported that dopamine, which in silico showed a comparable affinity for mammalian adrenergic receptors and QseC, had no effect on the activity of this kinase [54]. Nevertheless, antagonists of adrenergic and dopaminergic receptors are able to block the response to catecholamines in bacteria [53,55]. Thus, it is likely that bacteria have NM-specific receptor systems, although their mechanisms of signal processing remain to be understood.

Catecholamines contain a catechol moiety similar to bacterial siderophores and can act as pseudosiderophores [31,56,57,58]. By interacting with iron retained by host transferrin or lactoferrin, all three catecholamines can transfer it to bacterial siderophores such as enterobactin [59,60,61,62]. Some bacteria have specialized transport systems adapted to catecholamine-mediated iron uptake [63,64]. By providing bacteria with extra access to iron, this interdomain connection can also provoke oxidative stress with the activation of stress resistance genes *sodA* and *oxyR* [34]. Therefore, not all changes caused by catecholamines in bacterial transcriptomes and proteomes are due to their direct effect on gene expression.

To affect gene expression directly, NMs must at least enter bacterial cells. To date, only two types of experimental data support this ability. First, Lyte and Brown [65] used the fluorophores IDT307 and ASP+, which are analogs of dopamine, norepinephrine, and serotonin, and showed their appearance in biofilms of *Lactobacillus salivarius*. Thus, at least some bacteria have homologs of eukaryotic transporters of monoamines (PMAT) and organic cations (ASP+). Second, the proteomic analysis of *E. coli* O127: H6 indicated the ability of the OmpA and OmpC porins to bind transferrins that interact with catecholamines. Since *ompA* mutagenesis impaired the ability of bacteria to respond to stress hormones, OmpA has been proposed as a potential entry point for catecholamines into bacterial cells [63]. Hence, the question of how bacteria can react to imported NMs has become relevant.

Since the regulatory mechanisms operating at the level of RNA synthesis are considered to be the most effective, and the functionality of many transcription factors depends on the interaction with effector molecules, it was reasonable to assume the ability of NMs to affect the expression of bacterial genes through interfering with natural ligands. Thus, the goal of our study was to assess the ability of five neuromodulators, the physical and chemical properties of which correspond to the effectors of many regulatory proteins, to interact with effector binding sites in the structure of bacterial transcription regulators.

## 2. Results

### 2.1. Structural Models of Neuromodulators and Transcription Factors Selected for Analysis

Models of the neurotransmitters epinephrine (Epi), norepinephrine (Nor), and dopamine (Dop), as well as the hormones melatonin (Mel) and serotonin (Ser), were selected for molecular docking based on the literature’s data testifying their participtions in host–microbiome communications, and 53 structural models of 22 *E. coli* transcription factors were used as potential targets for their binding (Table 1).

The main criteria for selecting the target proteins were the dependence of their regulatory function on interaction with effector molecules and the presence of ligand binding sites in available 3D models. Priority was given to transcription factors that, according to information obtained from specialized databases [66,67,68], are associated with metabolic/regulatory pathways potentially involved in host–microbe interactions. As a result, the set of analyzed proteins included regulators of genes, controlling motility, virulence, and the stress response of *E. coli*, as well as transcription factors responsible for the synthesis of essential amino acids and biofilm formation (Table 1). All 3D models used in the study represent proteins reconstructed from X-ray diffraction data. They were taken from the Protein Data Bank of Japan (PDBJ) [69] and prepared for analysis as described in Section 4. To assess the ability of NMs to interact with ligand binding sites attuned for a complex function, four transcription factors combining the regulatory role with enzymatic activity were added to the set. Three of them (AidB, BirA, and PutA) have binding sites for relatively large non-protein ligands, and the HicB antitoxin has an interface for interaction with the toxic peptide HicA [66].

### 2.2. The Interaction of Neuromodulators with the Local Transcription Factor LacI Was Predicted at the Site of IPTG Binding for All Studied Protein Models

LacI is a repressor of a single operon of *E. coli* (lacZYA), which encodes lactose utilization and transport enzymes. The natural effector of LacI is allolactose; however, isopropyl β-d-1-thiogalactopyranoside (IPTG), a molecular mimic of the allolactose, is routinely used as a full-fledged functional analog. The protein models selected for docking (Table 2) were either dimers (1JYE and 1JYF) or tetramers (1TLF, 3EDC, and 4RZS) with a deletion of the first 60–61 amino acid residues in four proteins except 4RZS. These deletions exclude the possibility of LacI interaction with DNA but do not affect its ability to bind the ligand and were, therefore, acceptable for molecular docking in this study. Among the available structures, only 1TLF contained IPTG (one molecule per monomer), and the 3EDC model was reconstructed for the LacI co-crystalized with a flexible hexane-1,6-diol molecule at the allolactose binding site. The remaining models represent the 3D structures of apoproteins.

**Figure 1:**
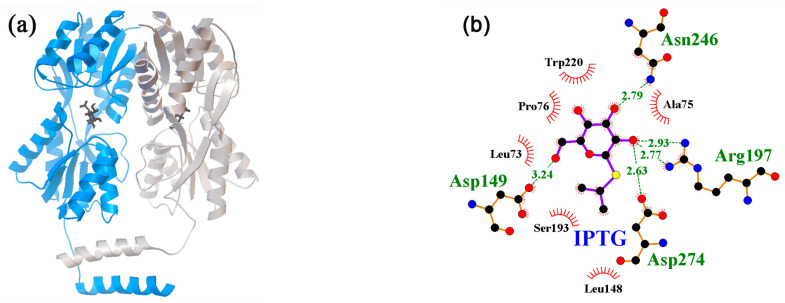
Structure of LacI complexes with IPTG reconstructed in the 1TLF model. (**a**) Three-dimensional structure of the LacI dimer (part of the 1TLF tetramer) complexed with IPTG. Two monomers of the protein are shown in blue and gray, while ligand molecules attached to monomers are black. (**b**) Ligand–protein interaction diagram obtained via LigPlot+ (v.2.2.8) [74] for an IPTG molecule bound to the inducer-binding center of LacI. Hereinafter, the green color indicates the amino acid residues that form hydrogen bonds with the ligand and the length of the H-bonds.

**Figure 2:**
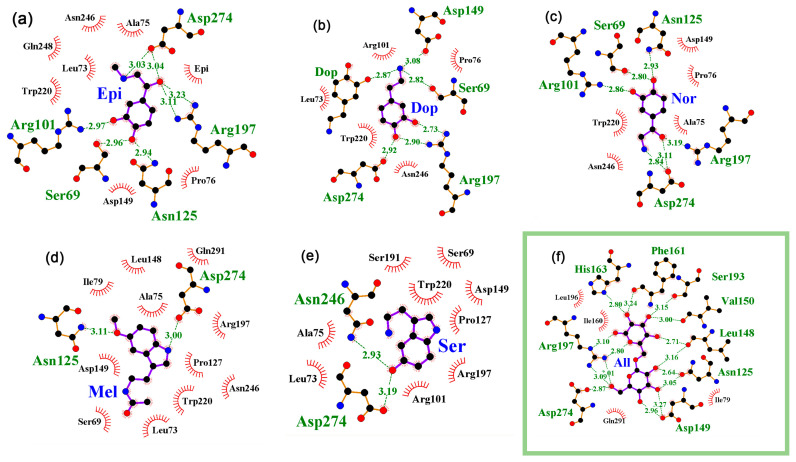
Predicted topology of interaction for neuromodulator molecules (**a**–**e**) and allolactose (**f**) with the ligand binding site of LacI in models 1TLF (**a**–**e**) and 3EDC, which showed the highest affinity to allolactose (**f**). H-bonds predicted in the local environment are marked as indicated for Figure 1b.

Molecular docking was performed for dimers of 1TLF, 3EDC, and 4RZS, while the 1JYE and 1JYF models were analyzed in monomeric forms provided by PDBJ. The binding of neuromodulators was predicted for all five structural models, and the region of interaction with the site occupied by IPTG (Figure 1) turned out to be the priority place of binding (Figure 2a–e). For epinephrine, norepinephrine, and dopamine, the predicted affinities of interaction were −6.8 (−5.3, ∆1.5) kcal/mol, −6.4 (−5.3, ∆1.1), and −6.3 (−5.3, ∆1.0) kcal/mol, respectively. The values in parentheses show the affinity of the neuromodulator molecule that most effectively interacted with the protein outside the IPTG binding site in the same docking iteration, and ∆ is the difference between site-specific and nonspecific binding. The “heat map” in the last five columns of Table 2 schematically shows the predicted specificity (∆) of the neuromodulators’ interaction with all protein models.

Interaction of melatonin with 1TLF model was the most efficient and specific (−7.9, ∆2.1 kcal/mol). Serotonin bound this model with lower predicted efficiency (−6.7, ∆1.4 kcal/mol) but reached −7.3 (∆2.0) kcal/mol for mutant protein 1JYE with K84A substitution outside the binding site (Table 2). Since the maximal affinity of the larger allolactose molecule (Figure 2f) for the effector site of LacI turned out to be only slightly higher (−9.2, ∆2.4 kcal/mol), the interaction of LacI with considered neuromodulators seems to be quite possible.

The amino acid residues that formed hydrogen bonds with IPTG in the 1TLF model were Asp274, Asp149, Arg197, and Asn246 (Figure 1b). Neuromodulators formed H-bonds predominantly with the same amino acids (Figure 2), and for Asp274, they were predicted in complexes with all NMs (Figure 2a–e). Although such interaction with Arg197 was predicted only for catecholamines (Figure 2a–c), additional bonds with Ser69, Arg101, and Asn125 can also be made by NMs in the effector binding site of LacI (Figure 2a–c). The estimated affinities of catecholamines for the IPTG binding site were lower than those of hormones, but in the predicted complexes, they formed more H-bonds, the number of which approached or even exceeded the number of bonds formed by the protein with IPTG (Figure 1b). In particular, if five H-bonds were observed in the crystal structure with IPTG, then the docking predicted six H-bonds for dopamine (Figure 2b), and even more can be formed by epinephrine and norepinephrine (Figure 2a,c). Thus, by occupying the ligand binding site, catecholamines can sterically hinder the interaction of the protein with the natural effector and limit the conformational mobility of LacI due to the formation of a branched network of H-bonds.

### 2.3. A Specific Interaction of at Least one Neuromodulator with the cAMP Binding Site Is Predicted for All Thirteen 3D Models of CRP

**Figure 3:**
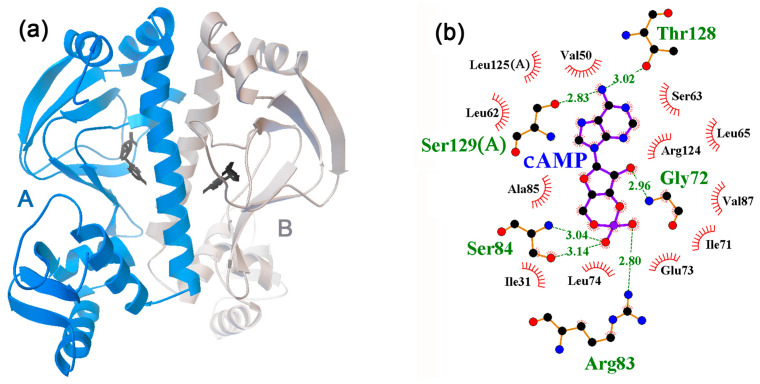
(**a**) Structure of the cAMP–CRP complex reconstructed for the 4I09 dimer and colored as indicated for Figure 1a. (**b**) Ligand–protein interaction diagram with monomer B. The amino acids belonging to monomer A are indicated in parentheses. Amino acid residues and H-bonds predicted in the local environment are marked as indicated for Figure 1b.

CRP is one of the global transcription regulators, which, according to the RegulonDB [66] and UniProt [67] databases, controls the expression of more than 600 genes (Table 1). The protein functions as a dimer (Figure 3a), and the character of its activity depends on cyclic AMP (cAMP) [75,76]. Twelve CRP models selected for docking were reconstructed for cAMP-CRP complexes with one effector molecule per monomer (Figure 3b), while the 4R8H model contained a cAMP analog in the ligand binding site (Table 3).

**Figure 4:**
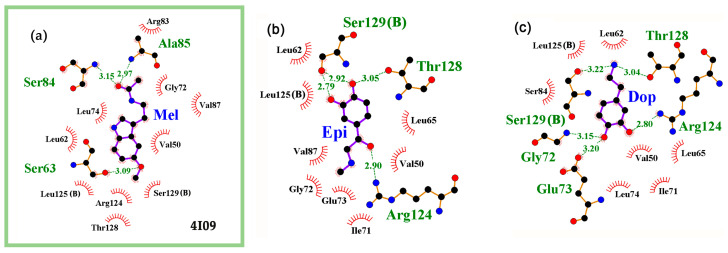

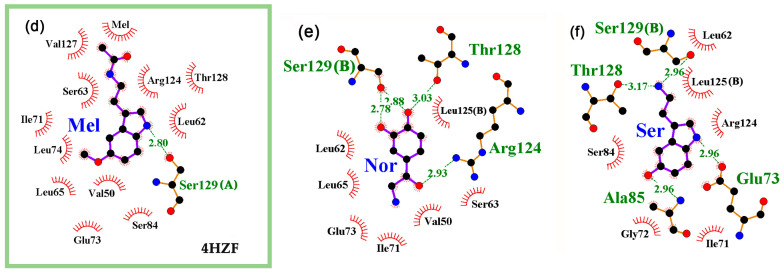
Predicted topology of interaction for neuromodulator molecules within the ligand binding site of CRP in the models 4I09 (**a**–**c**,**e**,**f**) and 4HZF (**d**). In model 4I09, most contacts with NMs were located in monomer A, whereas in model 4HZF, they predominantly interacted with monomer B. Therefore, the belonging of Ser129 and Leu125 to another subunit is indicated in parentheses. H-bonds in the local environments are marked as indicated for Figure 1b.

The amino acid residues that formed H-bonds with cAMP were Gly72, Arg83, Ser84, Ser129, and Thr128 (Figure 3b), which was consistent with the mutational analysis of the cAMP binding site in Crp [85]. We found that the preferential interaction of at least one neuromodulator with this site is possible for all protein models, whereas 1CGP, 1I5Z, 1ZRF, and 4R8H specifically bound all potential ligands (Table 3). The greatest variability was predicted for the 4I09 model, which specific affinity was minimal for dopamine (5.8, ∆0.1 kcal/mol) and maximal for melatonin (7.6, ∆2.4 kcal/mol) (Table 3). However, estimates made for the 4I0B and 4HZF 3D models reconstructed in the same study [83], predicting approximately the same affinity/specificity for epinephrine, norepinephrine, and serotonin as for the 4I09 model, showed great differences between the models for dopamine and melatonin (marked in Table 3). The dependence of docking results on the structural models could be traced for all studied NMs. Although the obvious differences between the 4HZF, 4I0B, and 4I09 models may have been due to the H160L and V132L substitutions near but outside the binding site (Figure 4a,d for 4I09 and 4HZF), the data obtained indicate the expediency of using several structural models for predictive docking.

Despite the fact that, in complexes with 4I09, the highest affinity and specificity was predicted for melatonin, it was the least active in forming H-bonds with CRP (Figure 4a–c,e,f) and in some cases did not form them at all. Dopamine and norepinephrine, and to a lesser extent epinephrine and serotonin, typically formed three or four such bonds, mainly with the same amino acid residues as cAMP (Figure 4b,c,e,f, respectively). Although this is fewer than the six H-bonds registered for cAMP (Figure 3b), the resulting complexes can be quite stable. Thus, it is likely that neuromodulators can successfully compete with cAMP for interaction with CRP. However, none of them turned out to be a complete analog of cAMP in terms of the local topology of the formed H-bonds (Figure 3b and Figure 4). Therefore, their ability to cause conformational rearrangements in the protein molecule necessary for the implementation of regulatory function seems doubtful.

### 2.4. Interaction of Neuromodulators with Effector Binding Sites of Bifunctional Proteins

Four transcription factors combining regulatory function with enzymatic activity were chosen with the aim of evaluating the ability of NM to interact with large ligand binding sites tuned to a complex function in which the probability of the random presence of a suitable locus for their binding is higher than when interacting with sites tuned to small effectors (Table 4). Two of them (AidB and PutA) have flavin adenine dinucleotide (FAD) as a cofactor, which made it possible to compare the preference of neuromodulators’ interaction with the binding site of the same ligand, but in different protein environments.

**Figure 5:**
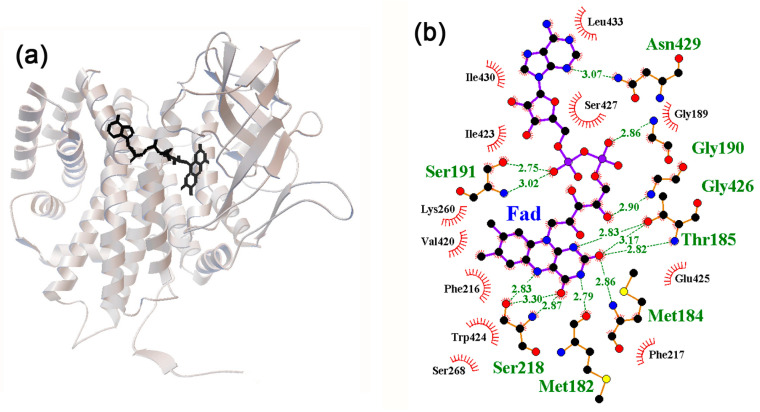
(**a**) Structure of the FAD–AidB complex reconstructed for the 3DJL monomer. (**b**) FAD–protein interaction diagram. H-bonds predicted in the local environment are marked as indicated for Figure 1b.

AidB is a repressor of its own gene [95] and has weak acyl–CoA dehydrogenase activity [96] (Table 1). The catalytic center and DNA-binding module of AidB are located in different domains. The reconstructed AidB tetramers contain flavin adenine dinucleotide (FAD) in the catalytic domain (Figure 5a), which promotes the assembly of the protein from dimers into tetramers or higher-order oligomers [87] and may participate in protecting the genome from approaching DNA alkylating agents [86].

**Figure 6:**
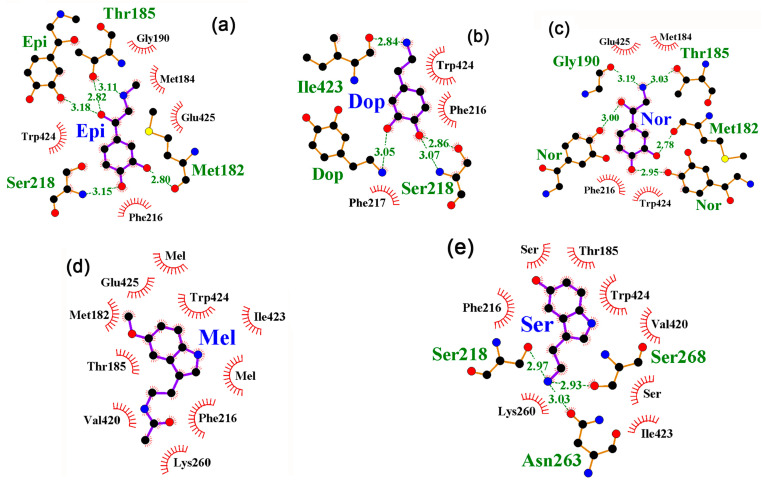
(**a**–**e**) Predicted topology of interaction for neuromodulator molecules within the ligand binding site of AidB model 3DJL. H-bonds in the local environments are marked as indicated for Figure 1b.

The amino acid residues involved in the interaction with FAD were Ser218, Met182, Met184, Thr185, Gly426, Gly190, and Ser191 (Figure 5b). Complexes with neuromodulators were predominantly formed with Ser218, Met182, and Thr185 (Figure 6). The remaining amino acid residues interacted with potential ligands episodically, but several NM molecules could simultaneously or sequentially occupy the FAD binding region, forming H-bonds with each other (Figure 6a–c). In the case of the 3DJL model, specific interaction involving H-bonds was predicted for epinephrine, dopamine, and serotonin (binding affinities: −7.2 (∆1.0), −6.3 (∆0.6), and −7.3 (∆1.4) kcal/mol, respectively) (Figure 6a,b,e). Norepinephrine interacted nonspecifically with the FAD binding site (−6.2, ∆0.1 kcal/mol), while melatonin, exhibiting high affinity and specificity for this region (−7.4, ∆1.3 kcal/mol), did not form hydrogen bonds at all (Figure 6d). Thus, the significantly larger area of the FAD binding region compared to that of cAMP and IPTG did not provide the presence of sites for specific interaction with all neuromodulators and did not guarantee the formation of H-bonds with them.

The PutA monomer (Table 1 and Table 4) is a polyfunctional flavoprotein that performs mutually exclusive functions, depending on the presence of proline and the redox state of FAD [97]. In the presence of proline, PutA acts as a bifunctional membrane enzyme catalyzing its oxidation by proline dehydrogenase and the subsequent oxidation of the product to glutamate by pyrroline-5-carboxylate dehydrogenase. In the absence of proline, PutA operates as a transcriptional repressor of the proline utilization genes [98]. Only one of three selected protein models (2FZN) contained proline in the X-ray structure, but all three models were co-crystallized with FAD (Table 4), the binding site of which was considered a specific area for interaction with neuromodulators.

The number of amino acid residues interacting with FAD in crystallized complexes was twice as large (Lys329, Ala371, Gln404, Arg431, Lys434, Gly435, Ala436, Thr457, Lys459, Thr462, His487, Thr486, Asn488, Arg555, Arg556, and Phe566) [93,94] as that in FAD complexes with AidB (Figure 5b and [86,87]). For all three catecholamines, specific interaction with this site was predicted for the models 2FZN and 3E2Q, but the binding of norepinephrine with the FAD binding site in model 4YNZ was nonspecific (Table 4). The highest affinity to the chosen neuromodulators demonstrated the proline-containing model 2FZN, with which melatonin formed a highly specific complex (−7.5, ∆2.5 kcal/mol). As in the case of CRP (model 4I09), the number of predicted hydrogen bonds for this hormone was found to be less than that of other neuromodulators, some of which formed higher-order complexes, as those shown for catecholamines in the FAD binding site of AidB (Figure 6a–c). Dopamine, for example, could simultaneously occupy this region with four molecules, forming up to five H-bonds each and ultimately interacting with most of the amino acid residues complexed with FAD (Lys329, Ala371, Arg431, Gly435, Lys459, Thr462, Thr486, His487, Asn488, Arg555, and Arg556), as well as with the neighboring Ala485, Gln511, Thr564, and Ser565. Therefore, the occupancy of the FAD site can be increased via the cooperative binding of several neuromodulator molecules, but as in the case of AidB, in the large area of the FAD binding center containing all types of amino acids for interaction, there was no place for the specific binding of norepinephrine in the 4JNZ model. Nevertheless, serotonin and melatonin showed a specific affinity for the FAD binding sites in both proteins, and in the cases of the 3DJL (AidB) and 4JNZ (PutA) models, the pattern of predicted interaction turned out to be similar (Table 4).

Perhaps the most compelling evidence against the random binding of neuromodulators to functionally important protein loci was obtained via analyzing the binding interface of the antitoxin HicB with the toxic peptide HicA. Acting as a transcription factor, HicB regulates the operon encoding the HicA–HicB type II toxin-antitoxin system and is involved in the *E. coli* envelope stress response, which could potentially be regulated by neuromodulators. However, over the entire surface of the apo form of HicB, not even a preferred binding site for any of the neuromodulators was found (Table 4).

**Figure 7:**
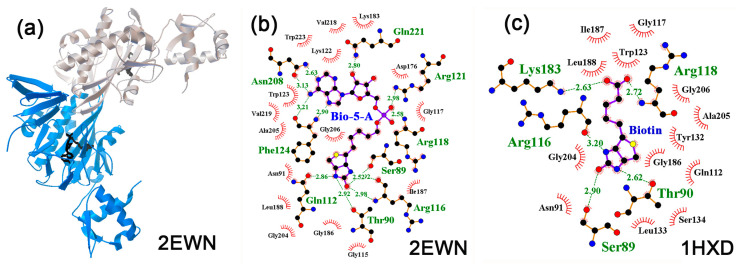
(**a**) Structure of the BirA in complex with effector biotinol-5AMP, reconstructed for the 2EWN dimer. (**b**,**c**) Interaction diagrams for biotinol-5AMP (**b**) and biotin (**c**) in the effector binding centers of the indicated protein models. H-bonds predicted in the local environment are marked as indicated for Figure 1b.

The bifunctional protein BirA exhibits biotin ligase activity and functions as a repressor of the biotin operon, depending on the presence of biotinol-5’-adenylate (bio-5′-AMP). Predominantly operating as a monomer, it was also crystallized as a dimer (Figure 7a), and it has been shown that bio-5’-AMP binding and dimerization are allosterically coupled [91]. Therefore, for both the monomer and dimer forms of BirA, crystal structures bearing either biotin or biotinol-5’-adenylate were used for molecular docking. As a result, estimates of the effectiveness of interaction for NMs on different models varied greatly. For the 1BIB monomer crystallized with biotin, a specific interaction was predicted only for melatonin and serotonin, while for the 4WF2 monomer crystallized with biotinol-5AMP, at least low-specific complexes may be formed with epinephrine, dopamine, and serotonin (Table 4).

**Figure 8:**
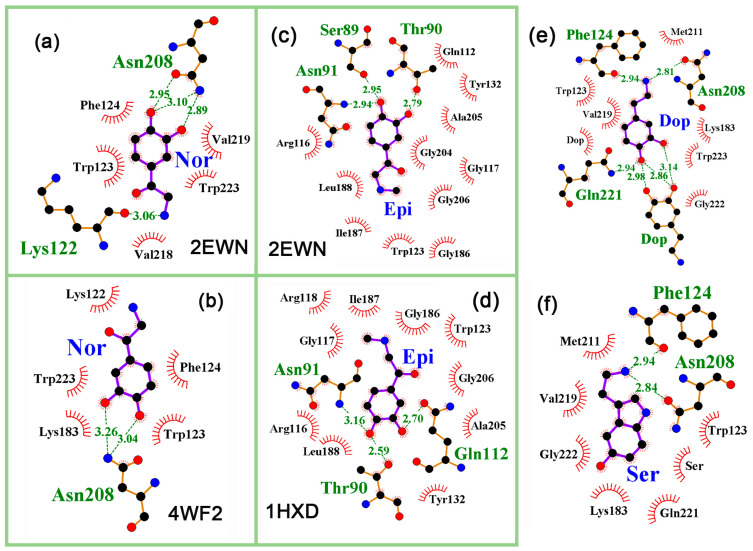
Predicted topology of neuromodulators binding with effector binding site of BirA. (**a**,**b**) Interaction of norepinephrine with biotinol-5AMP binding sites in 2EWN (**a**) and 4WF2 (**b**) protein models. (**c**,**d**) Interaction of epinephrine with biotinol-5AMP binding sites in 2EWN (**c**) and biotin binding locus in 1HXD (**d**) protein models. (**e**,**f**) Interaction of dopamine (**e**) and serotonin (**f**) with effector binding site in 2EWN protein model. H-bonds in the local environments are marked as indicated for Figure 1b.

In the complex reconstructed for the 2EWN dimer, biotinol-5AMP made contacts with many amino acid residues, including Gln221, Arg121, Arg118, Ser89, Arg116, Thr90, Gln112, Phe124, and Asn208 (Figure 7b). Five of them were predicted to form H-bonds with at least one potential ligand (Figure 8a,c,e,f), and the 2EWN dimer demonstrated the ability to bind all NMs tested (Table 4). The 4WF2 model differs from the 2EWN model in its monomeric state and the G142A mutation near the effector binding site. Since the G142A substitution abolishes dimerization/ligand binding coupling [99], both may cause structural rearrangements in the contact area, reducing the predicted binding efficiency of the 4WF2 model. For norepinephrine, it decreased from −6.5 (Δ1.0) kcal/mol to −5.6 (Δ0.6) kcal/mol with clearly visible rearrangements in the local environment (Figure 8a,b).

However, the largest difference in the predicted binding efficiencies was obtained for two dimeric models, 2EWN and 1HXD, differing in the co-crystallized ligands (biotinol-5AMP and biotin, respectively). In the 1HXD dimer, almost the same set of amino acids as in 2EWN (Ser89, Thr90, Arg118, Arg119, and Lys183) formed H-bonds with biotin (Figure 7c), and a clearly specific interaction with epinephrine (−7.2, Δ2.4 kcal/mol, Figure 8d) was predicted at the site, for which no other neuromodulator had a specific affinity (Table 4).

In the case of 2EWN protein model, the interaction with epinephrine was also specific (−7.7 (Δ1.7) kcal/mol, Figure 8c), but the ability to interact with the biotinol-5AMP binding site for all other NMs was also predicted (Table 4) and varied in the range of −6.2 (Δ0.8) kcal/mol (melatonin) to −6.9 (Δ1.2) kcal/mol (dopamine). Besides the inevitable experimental variations, such a large difference between the two docking profiles can be explained through different conformational changes of the allosteric center induced via different ligands co-crystalized with BirA (Figure 7b,c).

### 2.5. Overall Assessment of the “Propensity” of Bacterial Transcription Factors to Bind NM

The variety of physiological effects caused by eukaryotic signal molecules suggests that bacteria have many targets for them among regulatory proteins. Their complete screening does not seem appropriate, but the available models allow us to assess the degree of propensity of bacterial transcription factors to interact with eukaryotic neuromodulators. In addition to the transcription factors already discussed above, 16 regulators with ligand-dependent activity were used in this study. All of them have natural ligands or their analogs in the effector binding sites [66], including the nickel assimilation gene regulator NikR, for which this role is played by specifically bound nickel ions, and DnaR, in which the co-effector ADP marks the binding interface of the effector protein DhaL (Table 5).

At least two structural models represented seven proteins of the last set. For NikR, regulating genes of the nickel uptake, the interaction was registered in at least one of the two models for all hormones, while complexes with MetJ, which controls methionine transport and biosynthesis genes were not predicted only for melatonin in the 1MJO model carrying the Q44K mutation (Table 5). Of particular importance may be the ability of neuromodulators to form complexes with LsrR. This repressor controls the expression of bacterial stress response operons, including the genes for quorum sensing and the capture of the AI-2 autoinducer (Table 1). Being a component of the quorum sensing system AI-2 exploits the receptor-mediated entrance to the bacterial cells [57], which is considered a signal transduction pathway for neuromodulators [58]. For transcription factors PspF, PurR, and TrpR, the interaction was predicted sporadically, although one of the three PurR models (2PUC) showed a high affinity for epinephrine (Table 5), and none of the tested NMs had a specific affinity for the effector binding site of RutR. It is noteworthy that, for RutR, PspF, and PurR, the difference in the affinity of interaction with NMs in the region of natural ligand binding was sometimes even lower than with other sites on the protein surface, or specific complexes formed later (not in the first iteration of docking) than complexes with other sites.

Nine proteins used in our study were represented by only one structural model (Table 5). For AsnC, CpxR, DhaR, and IclR, no specific interaction with the binding sites of natural ligands was predicted. It may be symptomatic that the 4LRZ DhaR model contains ADP as a co-effector, for the binding site of which, in the PspF E108Q model, the neuromodulators studied also had no affinity (Table 5). Melatonin showed some preference to the binding site of sialic acid isomers in NanR, which are important signaling molecules used by bacteria for cell recognition during host colonization [109]. Epinephrine and norepinephrine interacted with the β-D-fructofuranose binding site of AscG, which controls two operons with genes involved in β-glucoside sugar transport/utilization and propionate catabolism. Three neuromodulators (epinephrine, melatonin, and serotonin) showed a capability for the specific interaction with TreR, for which the natural ligand trehalose-6-phosphate, in many species, acts as a messenger in signal transduction [117]. Four studied neuromodulators, excluding epinephrine, interacted with the L-arginine binding site in ArgR, which has a very large regulon with arginine biosynthesis and uptake genes. Finally, only serotonin did not interact with the acyl–CoA binding site of the global lipid metabolism regulator FadR.

Taken together, the molecular docking results showed that not all transcription factors have the ability to specifically interact with neuromodulators, and none of those that are able to do so showed a significantly higher affinity for eukaryotic signaling molecules compared to bacterial effectors. Nevertheless, the data obtained indicate that there is a way in which direct signal transmission from the host to the bacterial transcriptional machinery can take place, and there is a way for bacteria to sense habitats at the level of the cell regulatory networks.

## 3. Discussion

The aim of this study was to evaluate the potential ability of five neuromodulators to occupy the effector binding sites of bacterial transcription factors. The main result that we obtained using flexible molecular docking indicates that host signaling molecules are able to interact with many bacterial regulators, forming at least part of the hydrogen bonds that are involved in complex formation with the effectors of these proteins. The predicted complexes were highly variable in affinity and specificity, showing a clear preference for the effector binding sites of some proteins and even a complete lack of preferential binding across the entire surfaces of others. This variability, obviously, could be due to several technical and biological reasons, including crystallization conditions, the presence of mutations near the effector binding sites, and the oligomeric state of crystallized protein. However, by observing the complexes formed by 5 neuromodulators with 53 protein models over 10 consecutive docking steps, we came to the general conclusion that not every protein can specifically bind eukaryotic signaling molecules. That means that those transcription factors that have a distribution of functional groups suitable for such interaction can be evolutionarily adapted to the perception of host regulatory signals.

One of the seemingly unexpected observations made in the study was the high variability in the predicted affinity of some neuromodulators for different models of the same transcription factors without obvious differences in their structure. In some cases (models 4FT8 and 4HZF of CRP or 3DJL and 3U33 of AidB), this can be explained by the presence/absence of different ions in the crystal structure, which can influence the local topology of the target region. However, an even more significant contribution to these differences may be made by conformational changes in effector binding sites induced by different ligands co-crystallized with target proteins. Thus, for example, the topology of the H-bonds formed by biotinol-5AMP and biotin in the 1EWN and 1HXD models of BirA, although similar, was not identical, at least not in terms of the contact made with Glu221 and Lys183 (Figure 7b,c). The virtual removal of co-crystalized effectors from the protein structure left ligand-induced conformational changes in the model. In some cases, the “closed” conformation of the effector site obtained in this way complicated the interaction even with natural ligands. Although this can be considered as a limitation of our approach, which obligatorily required X-ray structural data on protein–ligand complexes, accurate information about the contacts formed with effector molecules made it possible to assess not only the binding affinity of the studied NMs but also the specificity of their interaction with the regulatory region of many proteins.

Intuitively, it is expected that proteins with large effectors containing heterocyclic components may be favorable targets for neuromodulator-mediated regulation. Thus, in the case of transcription factors with enzymatic activity AidB and PutA, FAD binding requires a surface area of 356 Å^2^, where there is room for several neuromodulators. However, the large areas of ligand binding sites in at least three bifunctional proteins, AidB, BirA, and HicB (Table 4), as well as in the transcription factor NanR (Table 5), did not demonstrate a clear advantage for specific interaction with the studied neuromodulators. Therefore, the degree of similarity/dissimilarity in the spatial organization of neuromodulators and natural effectors was considered a more important factor determining the specificity of the interaction of NMs with target proteins. According to PubChem [118], the topological polar surface area (TPSA) of cAMP is 155 Å^2^, and the interaction surface on the protein is adapted to bind a “rigid” molecule containing heterocyclic components. Consequently, catecholamines, as well as tryptophan derivatives with heterocycles and a flat surface with TPSA ranging from 54.1 Å^2^ (melatonin) to 86.7 Å^2^ (norepinephrine), are well suited for interaction with the cAMP binding site of CRP. If this is so, then the specific binding of neuromodulators could be expected with the effector sites of MetJ and PspF, which turned out to be correct only for MetJ (Table 5). This probably indicates that other physicochemical features of the native ligand also make a significant contribution to the specificity of the interaction.

The priority of neuromodulator interaction with proteins that perform some specific cellular functions was not explicitly traced, but it was not excluded either. It may be particularly expedient for systems involved in interaction with the host, including the bacterial production or modification of vital metabolites, such as amino acids, which cannot be produced in vertebrates, vitamins, and some carbon sources [119,120,121]. Methionine, for example, is an essential amino acid produced by fungi and bacteria that is necessary for protein synthesis in all biological objects. Beyond being the first amino acid encoded in a polypeptide, it is used by mammals in many regulatory events [122] and serves as a precursor for the excitatory neuromodulator homocysteine [121]. The repressor activity of MetJ depends on the interaction with the effector S-adenosyl methionine (AdoMet), which inhibits excessive methionine biosynthesis in *E. coli* and plays a crucial role in many regulatory processes as a universal donor of methyl groups. By interacting with MetJ, AdoMet creates additional electrostatic contacts with the phosphodiester backbone of DNA, which significantly increases the affinity of MetJ for operator sites [123,124]. Here, we have shown that neuromodulators have a relatively high specificity for the AdoMet-binding site of MetJ (Table 5). Their presence, therefore, may stimulate methionine synthesis by reducing the repressor activity of MetJ.

At least the low-specific binding predicted for the interaction of catecholamines with TrpR (Table 5) may have the same stimulating effect not only on the biosynthesis of tryptophan, which is a precursor to serotonin, but also on the production of phenylalanine (the precursor to all catecholamines). However, in this case, the promoter-binding ability of TrpR homodimers is allosterically activated by two L-tryptophan molecules, which stabilize the classical helix-turn-helix DNA-binding motif in the orientation favorable for interaction with DNA [116,125]. By occupying the TrpR effector site, host signaling molecules can weaken its inhibitory effect. Even if the topology of their interaction with the transcription factor was not evolutionarily tuned for the induction of conformational transitions required for allosteric regulation, this may be sufficient to maintain essential amino acid production, which is mutually beneficial for the microbial consortium and the host.

For none of the proteins we considered was any such interaction with an exogenous ligand observed that would completely reproduce the binding topology of the natural effector. There was always some variability in the set of involved amino acid residues on the protein and in the distribution of hydrogen bonds between amino acid residues and the ligand molecules. Perhaps this means that the interaction of many studied proteins with neuromodulators is only competitive with respect to the native ligand reducing its functionality. However, in the case of LacI, the occupancy of the effector binding site itself may be of the highest biological significance. This transcription factor regulates the expression of only one, but metabolically a very important, operon of *E. coli* (lacZYA), which encodes a protein promoting the uptake of lactose (LacY) and two enzymes, of which β-galactosidase (LacZ) breaks down lactose into two metabolizable monosaccharides, glucose and galactose. In the absence of lactose, LacI strongly represses the transcription of lacZYA by sterically preventing open promoter complex formation with RNA polymerase. If lactose is available, its natural isomer allolactose relieves repression by occupying the DNA-binding site in LacI, thus interfering with its repressor function. Synthetic β-galactosides, including IPTG, as well as some other compounds, such as methyl-β-d-thiogalactopyranoside, can replace allolactose in this function [126]. It is, therefore, likely that neuromodulators that have demonstrated a high affinity and specificity for the LacI ligand binding site (Table 2) may also act as inducers of the lac operon.

Although lactose is the main energy source in milk, the production of an enzyme converting it into monosaccharides (lactase) in humans usually falls with age, which often leads to lactose intolerance. Therefore, the evolutionarily developed ability of host neuromodulators to activate lactose consumption by bacteria can be considered part of the “niche construction process” [127] carried out by mammals and their microbial communities. Since the data obtained from molecular docking are perfectly reproducible in the case of LacI (Table 2), we consider this transcription factor the best candidate for the experimental confirmation of the ability of NMs to influence the expression of bacterial genes. We believe that some other (perhaps many, but not all) bacterial proteins from our set may specifically recognize and respond to host signaling molecules, implementing binding patterns individually tailored for each transcription factor to tune its functional activity to the ecological niche provided by the host.

## 4. Materials and Methods

### 4.1. Structural Models of Neuromodulators Used in This Work

Three-dimensional models of dopamine, epinephrine, norepinephrine, serotonin, and melatonin were obtained from the PubChem database [118]. For molecular docking, they were prepared using the Avogadro program (v. 1.2.0.) [128].

### 4.2. Structural Models of Transcription Factors Used in This Work

Fifty-three structural models of 22 *E. coli* transcription factors were used as potential targets for interaction with structural models of neuromodulators (Table 1). All protein models were obtained via X-ray diffraction and reconstructed with resolution 1.42–3.0 Å. The PDB files were taken from the Protein Data Bank of Japan (PDBJ) database [69]. To estimate the affinity of the interaction between neuromodulators and the binding site of natural effectors, the ligand models present in the protein structure were virtually removed before docking.

### 4.3. Flexible Protein–Ligand Docking

Flexible molecular docking was carried out using the open-source program AutoDock VINA [129], and potentially motile bonds in the structure of each ligand flexible were retained. The calculated free energy accounted for various types of forces between atoms, including but not limited to van der Waals, electrostatic, hydrophilic, and hydrophobic interactions, hydrogen bonding, and desolvation. The strategy of the docking was almost the same as described previously [130,131]. For each ligand, preferential binding sites were determined, and affinity values were calculated. The ligand models with the highest affinity bound to the target model in each docking round were sequentially added to the structure, and the resulting complexes were used in the next docking round as a target. The docking of neuromodulators for each protein model included 10 consecutive iterations, each of which used the structure formed in the previous docking round, and the program searched for the binding site of the next ligand molecule. The affinity of the first neuromodulator molecule specificaly bound to the natural ligand site was considered a measure of specificity, even if this was not observed in the first round of successive iterations.

The specificity of the interaction was determined using the location of the neuromodulator molecule in the complex with the protein and its ability to form hydrogen bonds with the same amino acid residues as the native ligand. The estimated values of affinity in the sites of preferential binding and their differences from the affinity of interaction with the next preferred site on the protein surface within one iteration of docking were calculated. Specificity rating was carried out in accordance with the difference in the affinity values of the neuromodulator model forming the most stable complex in the binding site of the natural ligand and its affinity for the most preferred place located outside this site (∆). Complexes with ∆ ≥ 2 kcal/mol were considered highly specific. The docking results were visualized using AutoDock Tools in Python Molecular Viewer (v. 1.5.6) [132]. The prediction and visualization of potential hydrogen bonds in the complex of the protein model and predicted ligand models were carried out using LigPlot+ v.2.2.8 [132].

## Figures and Tables

**Table 1:** The list of transcription factors whose 3D models were chosen for molecular docking.

TF	Functional Category	Size of Regulon		N. of Models
		Operons	Genes	
Transcription factors				
ArgR	Biosynthesis of L-arginine (main intestinal metabolite)	16	64	1
AscG	Transport and assimilation of β-glucosides	4	5	1
AsnC	Asparagine biosynthesis	2	4	1
CpxR	Stress response	40	72	1
CRP	Global regulator of catabolite repression	274	625	13
DhaR	Activator of dihydroxyacetone kinase genes (detoxification)	2	4	1
FadR	Global regulator of lipid and fatty acid metabolism	17	23	1
LacI	Transport of lactose and its conversion into glucose and galactose	1	3	5
IclR	Control of a glyoxylate bypass operon upon acetate accumulation	2	4	1
LsrR	AI-2 uptake, stress response, host invasion, and biofilm formation	4	9	2
MetJ	Biosynthesis and transport of methionine (essential amino acid)	10	15	2
NanR	Sialic acid transport and assimilation (bacterial pathogenesis)	4	11	1
NikR	Nickel uptake (host-specific induction)	1	6	2
PspF	Stress and phage shock response	3	8	2
PurR	Purine biosynthesis	20	32	3
RutR	Pyrimidine metabolism	7	17	2
TreR	Trehalose transport/degradation (host colonization and virulence)	1	1	1
TrpR	Tryptophan and phenylalanine biosynthesis (essential amino acids)	5	10	3
	Transcription factors with enzymatic activity and enzymes with transcription regulatory function			
AidB	Isovaleryl-CoA dehydrogenase, resistance against alkylation agents	1	1	2
BirA	Biotin ligase (protein biotinilation), repressor of biotin synthesis	2	5	4
HicB	Antitoxin of HicA-HicB system, extracytoplasmic stress response	2	2	1
PutA	Proline/pyrroline-5-carboxylate dehydrogenase, oxidative stress response	2	2	3

**Table 2:** Relative affinity of interaction between five reconstructed 3D models of LacI and neuromodulators.

Model	Reference	Resolution (Å)	Ligand, Other Compounds	Mutations	Epi	Dop	Nor	Mel	Ser
1JYE	[70]	1.7	Glycerol	K84L A109T					
1JYF	[70]	3.0	Glycerol	A109T					
1TLF	[71]	2.6	IPTG, C_2_H_5_Hg						
3EDC	[72]	2.1	Hexane-1,6-diol						
4RZS	[73]	2.71	Glycerol	D152T V153A, I159L, S196D					

Specificity scale: black—complexes with ∆ ≥ 2 kcal/mol; dark gray—1.0 ≤ ∆ < 2 kcal/mol or if all complexes in the iteration were predicted at the effector binding site.

**Table 3:** Relative affinity of interaction between 3D models of CRP and neuromodulators.

Model	Reference	Resolution (Å)	Ligand, Other Compounds	Mut	Epi	Dop	Nor	Mel	Ser
1CGP	[77]	3.0	DNA, cAMP						
1G6N	[78]	2.1	cAMP						
1HW5	[79]	1.82	cAMP						
1I5Z	-	1.9	cAMP, triol						
1I6X	-	2.2	cAMP, triol	D54H					
1ZRF	[80]	2.1	DNA, cAMP and 1,4-dioxane						
3KCC	[81]	1.66	cAMP, glycerol	D138L					
3QOP	-	1.96	cAMP, glycerol						
4FT8	[82]	1.966	cAMP, SO_4_ and Co^2+^						
4HZF	[83]	1.48	cAMP, glycerol and HPO_4_^2−^						
4I0B	[83]	1.50	cAMP	H160L					
4I09	[83]	2.05	cAMP	V132L					
4R8H	[84]	1.46	RP-adenosine-3′,5′-cyclic-mono-phosphorothioate, glycerol						

Specificity scale: black—complexes with ∆ ≥ 2 kcal/mol; dark gray—1.0 ≤ ∆ < 2 kcal/mol or if all complexes in the iteration were predicted at the effector binding site; light gray—0.6 ≤ ∆ <1.0 kcal/mol; white cells correspond to ∆ < 0.6 kcal/mol. Red boxes highlight the variability in docking results obtained for dopamine and melatonin, which may be mediated by point mutations or crystallization conditions.

**Table 4:** Relative affinity of NM interaction with bifunctional transcription factors (specificity scale is the same as for Table 3).

Protein	Model	Reference	Resolu-tion (Å)	Ligand, Other Compounds	Mut	Epi	Dop	Nor	Mel	Ser
AidB	3DJL	[86]	1.7	FAD, Ca^2+^						
	3U33	[87]	2.8	FAD, Cl^−^						
BirA	1HXD	[88]	2.4	Biotin						
	1BIB	[89]	2.8	Biotin						
	2EWN	[90]	2.8	Biotinol-5AMP						
	4WF2	[91]	2.31	Biotinol-5AMP	G142A					
HicB	6HPC	[92]	2.26	-						
PutA	2FZN	-	2.0	FAD, proline						
	3E2Q	[93]	1.75	FAD, 4-hydroxyproline and pentaethyleneglycol	Y540S					
	4JNZ	[94]	1.85	FAD,4-hydrofuran-2-carboxylicacid and pentaethyleneglycol	D370N					

**Table 5:** Relative affinity/specificity of interaction between *E. coli* transcription factors and eukaryotic neuromodulators.

Protein	Model	Ref.	Resolu-tion (Å)	Effectors, Other Compounds	Mut	Epi	Dop	Nor	Mel	Ser
ArgR	1XXB	[100]	2.6	Arginine						
AscG	3BRQ	-	2.0	β-D-fructofuranose, SO_4_^2−^ and Na^+^						
AsnC	2CG4	[101]	2.4	Asparagine, Mg^2+^	G37E					
CpxR	4UHK	[102]	2.6	Phosphorylated protein, Mg^2+^						
DhaR	4LRZ	[103]	2.32	ADP (co-effector), Mg^2+^						
FadR	1H9G	[104]	2.1	Co-enzyme A, myristic acid						
IclR	2O99	[105]	1.7	1,2-Ethanediol, Glycolicacid	M*					
LsrR	4L4Z	[106]	2.3	(2S)-2,3,3-trihydroxy-4-oxopentyl dihydrogen phosphate						
	4L51	[106]	1.9	5-O-phosphono-alpha-D-ribofuranose						
MetJ	1CMC	[107]	1.8	S-Adenosylmethionine, Mg^2+^						
	1MJO	[108]	2.1	DNA, S-Adenosylmethionine, Ca^2+^	Q44L					
NanR	6ON4	[109]	2.1	N-acetyl-β-neuraminic acid, PEG, Zn^2+^						
NikR	2HZA	-	2.1	Ni^2+^ (effector), 3-Cyclohexylpropyl 4-O-α-D-gluco-pyranosyl-β-D-glucopyranoside	M**					
	3OD2	-	2.6							
PspF	2C9C	[110]	2.1	ATP (effector), Mg^2+^	R227A					
	4QOS	[111]	1.42	ADP (effector), glycerol and HEPES	E108Q					
PurR	1JFT	[112]	2.5	DNA, hypoxantine and PO_4_^3−^	W146A					
	1VPW	[113]	2.7	DNA, hypoxantine (effector)	L53M					
	2PUC	[114]	2.6	DNA, guanine (effector)	R189A					
RutR	3LOC	-	2.5	Uracil						
	4XK4	-	2.27	Dihydropyrimidine-2,4(1H,3H)-dione						
TreR	4XXH	[115]	2.4	Trehalose-6-phosphate						
TrpR	1TRO	[116]	1.9	DNA, tryptophan	Q13E					
	1ZT9	-	2.0	Tryptophan, SO_4_^2−^						
	6F7F	-	2.13	Indolylpropionic acid						

The specificity scale is the same as in Table 3. M* M113, M122, M144, M146, M156, M258, and M273 substituted with selenomethionine. M** M1 and M105 substituted with selenomethionine.

## Data Availability

Not applicable.
